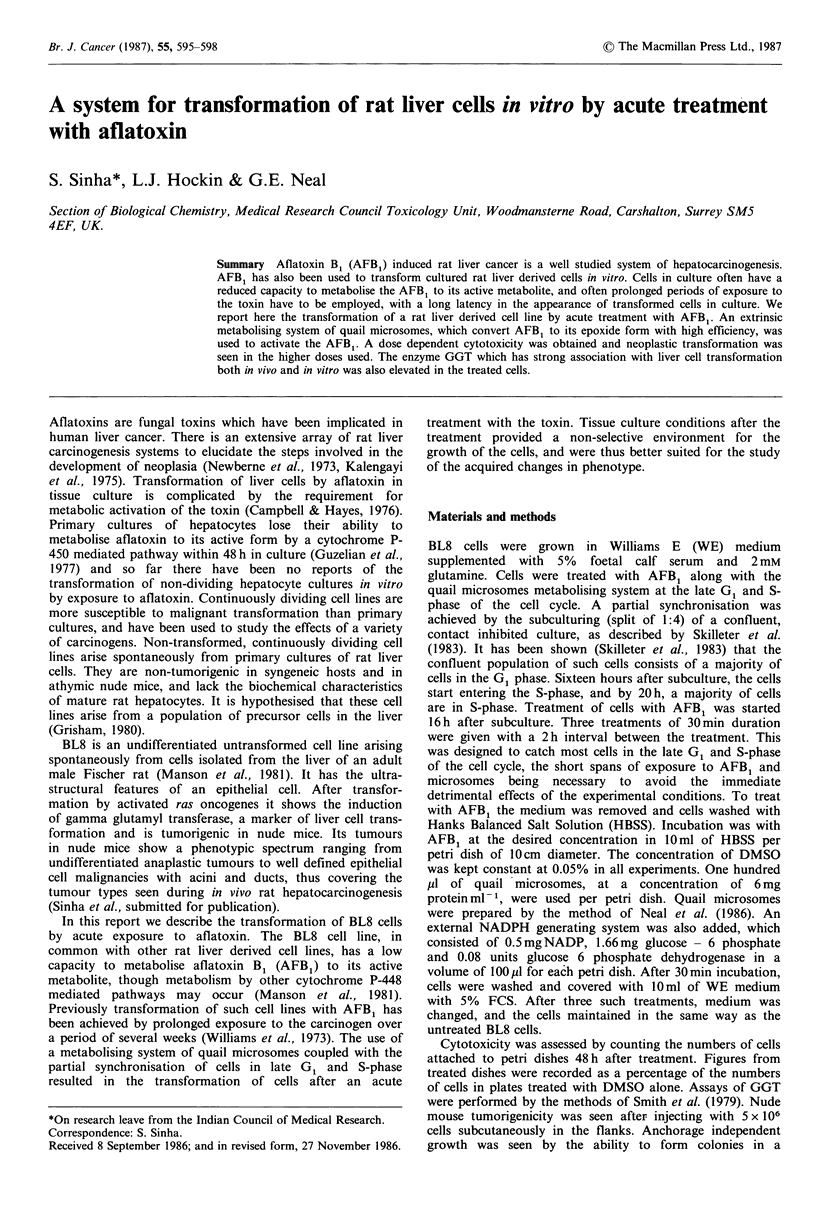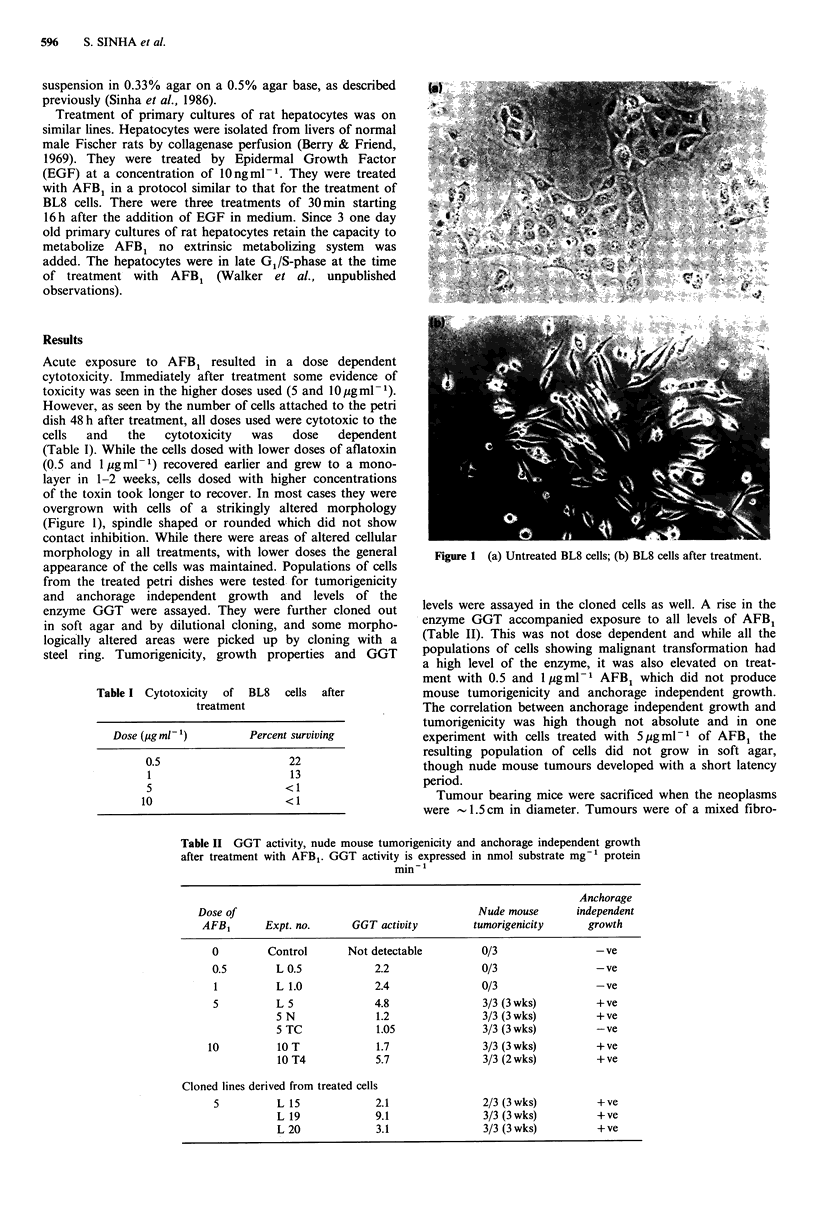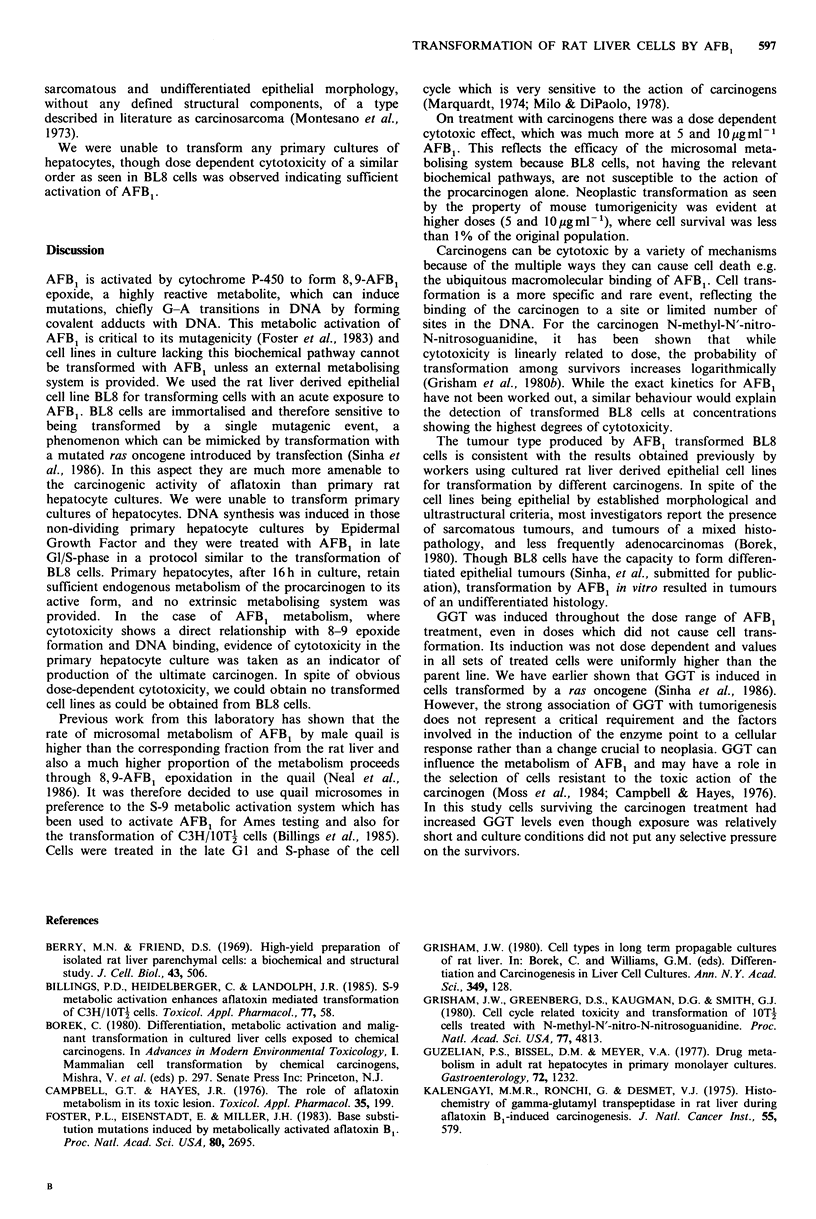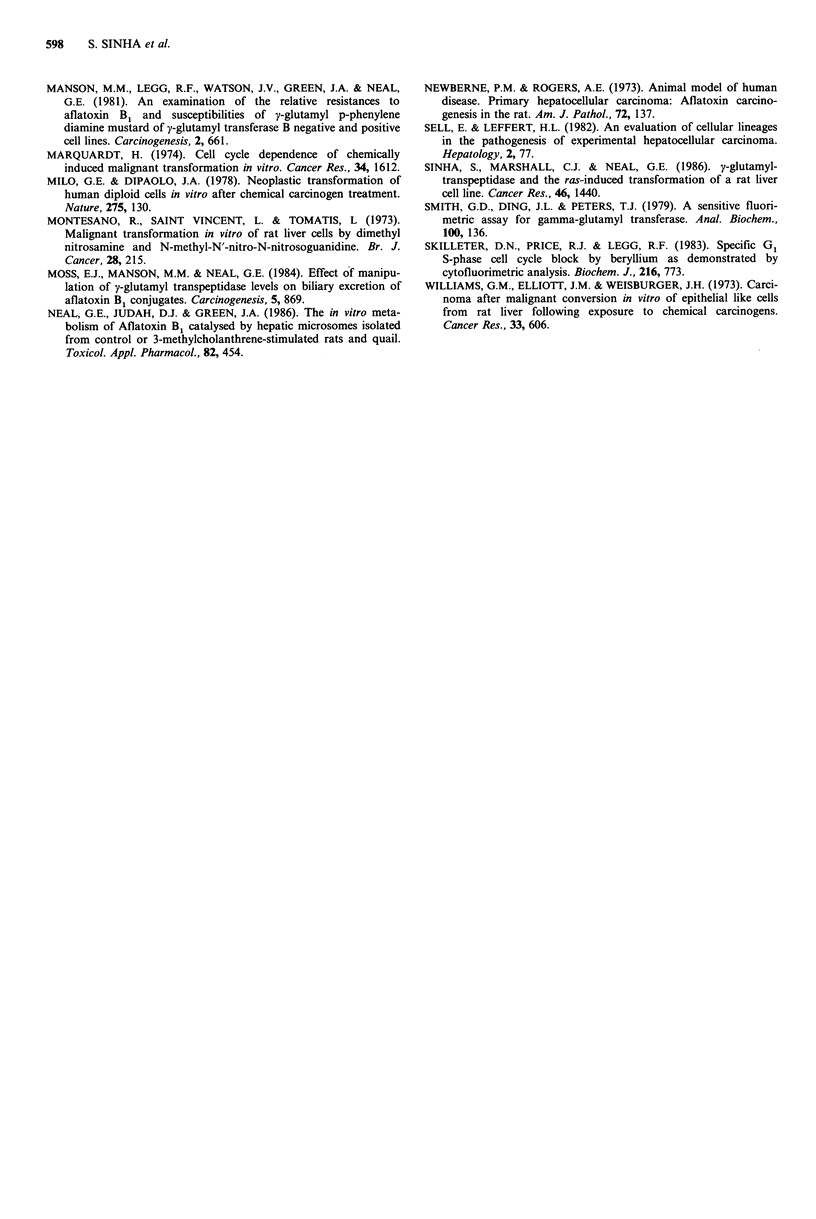# A system for transformation of rat liver cells in vitro by acute treatment with aflatoxin.

**DOI:** 10.1038/bjc.1987.121

**Published:** 1987-06

**Authors:** S. Sinha, L. J. Hockin, G. E. Neal

## Abstract

**Images:**


Br. J. Cancer (1987), 55, 595–598 © The Macmillan Press Ltd., 1987

### A system for transformation of rat liver cells *in vitro* by acute treatment with aflatoxin

S. Sinha*, L.J. Hockin & G.E. Neal

*Section of Biological Chemistry, Medical Research Council Toxicology Unit, Woodmansterne Road, Carshalton, Surrey SM5 4EF, UK.*

**Summary** Aflatoxin $B_1$ ($AFB_1$) induced rat liver cancer is a well studied system of hepatocarcinogenesis. $AFB_1$ has also been used to transform cultured rat liver derived cells *in vitro*. Cells in culture often have a reduced capacity to metabolise the $AFB_1$ to its active metabolite, and often prolonged periods of exposure to the toxin have to be employed, with a long latency in the appearance of transformed cells in culture. We report here the transformation of a rat liver derived cell line by acute treatment with $AFB_1$. An extrinsic metabolising system of quail microsomes, which convert $AFB_1$ to its epoxide form with high efficiency, was used to activate the $AFB_1$. A dose dependent cytotoxicity was obtained and neoplastic transformation was seen in the higher doses used. The enzyme GGT which has strong association with liver cell transformation both *in vivo* and *in vitro* was also elevated in the treated cells.

Aflatoxins are fungal toxins which have been implicated in human liver cancer. There is an extensive array of rat liver carcinogenesis systems to elucidate the steps involved in the development of neoplasia (Newberne *et al.*, 1973, Kalengayi *et al.*, 1975). Transformation of liver cells by aflatoxin in tissue culture is complicated by the requirement for metabolic activation of the toxin (Campbell & Hayes, 1976). Primary cultures of hepatocytes lose their ability to metabolise aflatoxin to its active form by a cytochrome P-450 mediated pathway within 48 h in culture (Guzelian *et al.*, 1977) and so far there have been no reports of the transformation of non-dividing hepatocyte cultures *in vitro* by exposure to aflatoxin. Continuously dividing cell lines are more susceptible to malignant transformation than primary cultures, and have been used to study the effects of a variety of carcinogens. Non-transformed, continuously dividing cell lines arise spontaneously from primary cultures of rat liver cells. They are non-tumorigenic in syngeneic hosts and in athymic nude mice, and lack the biochemical characteristics of mature rat hepatocytes. It is hypothesised that these cell lines arise from a population of precursor cells in the liver (Grisham, 1980).

BL8 is an undifferentiated untransformed cell line arising spontaneously from cells isolated from the liver of an adult male Fischer rat (Manson *et al.*, 1981). It has the ultra-structural features of an epithelial cell. After transformation by activated *ras* oncogenes it shows the induction of gamma glutamyl transferase, a marker of liver cell transformation and is tumorigenic in nude mice. Its tumours in nude mice show a phenotypic spectrum ranging from undifferentiated anaplastic tumours to well defined epithelial cell malignancies with acini and ducts, thus covering the tumour types seen during *in vivo* rat hepatocarcinogenesis (Sinha *et al.*, submitted for publication).

In this report we describe the transformation of BL8 cells by acute exposure to aflatoxin. The BL8 cell line, in common with other rat liver derived cell lines, has a low capacity to metabolise aflatoxin $B_1$ ($AFB_1$) to its active metabolite, though metabolism by other cytochrome P-448 mediated pathways may occur (Manson *et al.*, 1981). Previously transformation of such cell lines with $AFB_1$ has been achieved by prolonged exposure to the carcinogen over a period of several weeks (Williams *et al.*, 1973). The use of a metabolising system of quail microsomes coupled with the partial synchronisation of cells in late $G_1$ and S-phase resulted in the transformation of cells after an acute treatment with the toxin. Tissue culture conditions after the treatment provided a non-selective environment for the growth of the cells, and were thus better suited for the study of the acquired changes in phenotype.

### Materials and methods

BL8 cells were grown in Williams E (WE) medium supplemented with 5% foetal calf serum and 2 mM glutamine. Cells were treated with $AFB_1$ along with the quail microsomes metabolising system at the late $G_1$ and S-phase of the cell cycle. A partial synchronisation was achieved by the subculturing (split of 1:4) of a confluent, contact inhibited culture, as described by Skilleter *et al.* (1983). It has been shown (Skilleter *et al.*, 1983) that the confluent population of such cells consists of a majority of cells in the $G_1$ phase. Sixteen hours after subculture, the cells start entering the S-phase, and by 20 h, a majority of cells are in S-phase. Treatment of cells with $AFB_1$ was started 16 h after subculture. Three treatments of 30 min duration were given with a 2 h interval between the treatment. This was designed to catch most cells in the late $G_1$ and S-phase of the cell cycle, the short spans of exposure to $AFB_1$ and microsomes being necessary to avoid the immediate detrimental effects of the experimental conditions. To treat with $AFB_1$ the medium was removed and cells washed with Hanks Balanced Salt Solution (HBSS). Incubation was with $AFB_1$ at the desired concentration in 10 ml of HBSS per petri dish of 10 cm diameter. The concentration of DMSO was kept constant at 0.05% in all experiments. One hundred $\mu l$ of quail microsomes, at a concentration of 6 mg protein $ml^{-1}$, were used per petri dish. Quail microsomes were prepared by the method of Neal *et al.* (1986). An external NADPH generating system was also added, which consisted of 0.5 mg NADP, 1.66 mg glucose – 6 phosphate and 0.08 units glucose 6 phosphate dehydrogenase in a volume of 100 $\mu l$ for each petri dish. After 30 min incubation, cells were washed and covered with 10 ml of WE medium with 5% FCS. After three such treatments, medium was changed, and the cells maintained in the same way as the untreated BL8 cells.

Cytotoxicity was assessed by counting the numbers of cells attached to petri dishes 48 h after treatment. Figures from treated dishes were recorded as a percentage of the numbers of cells in plates treated with DMSO alone. Assays of GGT were performed by the methods of Smith *et al.* (1979). Nude mouse tumorigenicity was seen after injecting with $5 \times 10^6$ cells subcutaneously in the flanks. Anchorage independent growth was seen by the ability to form colonies in a

*On research leave from the Indian Council of Medical Research.
Correspondence: S. Sinha.
Received 8 September 1986; and in revised form, 27 November 1986.

suspension in 0.33% agar on a 0.5% agar base, as described previously (Sinha et al., 1986).

Treatment of primary cultures of rat hepatocytes was on similar lines. Hepatocytes were isolated from livers of normal male Fischer rats by collagenase perfusion (Berry & Friend, 1969). They were treated by Epidermal Growth Factor (EGF) at a concentration of $10\,ng\,ml^{-1}$. They were treated with $AFB_1$ in a protocol similar to that for the treatment of BL8 cells. There were three treatments of 30 min starting 16 h after the addition of EGF in medium. Since 3 one day old primary cultures of rat hepatocytes retain the capacity to metabolize $AFB_1$ no extrinsic metabolizing system was added. The hepatocytes were in late $G_1$/S-phase at the time of treatment with $AFB_1$ (Walker et al., unpublished observations).

### Results

Acute exposure to $AFB_1$ resulted in a dose dependent cytotoxicity. Immediately after treatment some evidence of toxicity was seen in the higher doses used (5 and $10\,\mu g\,ml^{-1}$). However, as seen by the number of cells attached to the petri dish 48 h after treatment, all doses used were cytotoxic to the cells and the cytotoxicity was dose dependent (Table I). While the cells dosed with lower doses of aflatoxin (0.5 and $1\,\mu g\,ml^{-1}$) recovered earlier and grew to a monolayer in 1–2 weeks, cells dosed with higher concentrations of the toxin took longer to recover. In most cases they were overgrown with cells of a strikingly altered morphology (Figure 1), spindle shaped or rounded which did not show contact inhibition. While there were areas of altered cellular morphology in all treatments, with lower doses the general appearance of the cells was maintained. Populations of cells from the treated petri dishes were tested for tumorigenicity and anchorage independent growth and levels of the enzyme GGT were assayed. They were further cloned out in soft agar and by dilutional cloning, and some morphologically altered areas were picked up by cloning with a steel ring. Tumorigenicity, growth properties and GGT levels were assayed in the cloned cells as well. A rise in the enzyme GGT accompanied exposure to all levels of $AFB_1$ (Table II). This was not dose dependent and while all the populations of cells showing malignant transformation had a high level of the enzyme, it was also elevated on treatment with 0.5 and $1\,\mu g\,ml^{-1}$ $AFB_1$ which did not produce mouse tumorigenicity and anchorage independent growth. The correlation between anchorage independent growth and tumorigenicity was high though not absolute and in one experiment with cells treated with $5\,\mu g\,ml^{-1}$ of $AFB_1$ the resulting population of cells did not grow in soft agar, though nude mouse tumours developed with a short latency period.

**Figure 1** (a) Untreated BL8 cells; (b) BL8 cells after treatment.

**Table I** Cytotoxicity of BL8 cells after treatment

| *Dose ($\mu g\,ml^{-1}$)* | *Percent surviving* |
|---|---|
| 0.5 | 22 |
| 1 | 13 |
| 5 | <1 |
| 10 | <1 |

Tumour bearing mice were sacrificed when the neoplasms were ~1.5 cm in diameter. Tumours were of a mixed fibro-

**Table II** GGT activity, nude mouse tumorigenicity and anchorage independent growth after treatment with $AFB_1$. GGT activity is expressed in nmol substrate $mg^{-1}$ protein $min^{-1}$

| *Dose of $AFB_1$* | *Expt. no.* | *GGT activity* | *Nude mouse tumorigenicity* | *Anchorage independent growth* |
|---|---|---|---|---|
| 0 | Control | Not detectable | 0/3 | −ve |
| 0.5 | L 0.5 | 2.2 | 0/3 | −ve |
| 1 | L 1.0 | 2.4 | 0/3 | −ve |
| 5 | L 5 | 4.8 | 3/3 (3 wks) | +ve |
| | 5 N | 1.2 | 3/3 (3 wks) | +ve |
| | 5 TC | 1.05 | 3/3 (3 wks) | −ve |
| 10 | 10 T | 1.7 | 3/3 (3 wks) | +ve |
| | 10 T4 | 5.7 | 3/3 (2 wks) | +ve |
| Cloned lines derived from treated cells | | | | |
| 5 | L 15 | 2.1 | 2/3 (3 wks) | +ve |
| | L 19 | 9.1 | 3/3 (3 wks) | +ve |
| | L 20 | 3.1 | 3/3 (3 wks) | +ve |

sarcomatous and undifferentiated epithelial morphology, without any defined structural components, of a type described in literature as carcinosarcoma (Montesano *et al.*, 1973).

We were unable to transform any primary cultures of hepatocytes, though dose dependent cytotoxicity of a similar order as seen in BL8 cells was observed indicating sufficient activation of $AFB_1$.

### Discussion

$AFB_1$ is activated by cytochrome P-450 to form 8,9-$AFB_1$ epoxide, a highly reactive metabolite, which can induce mutations, chiefly G–A transitions in DNA by forming covalent adducts with DNA. This metabolic activation of $AFB_1$ is critical to its mutagenicity (Foster *et al.*, 1983) and cell lines in culture lacking this biochemical pathway cannot be transformed with $AFB_1$ unless an external metabolising system is provided. We used the rat liver derived epithelial cell line BL8 for transforming cells with an acute exposure to $AFB_1$. BL8 cells are immortalised and therefore sensitive to being transformed by a single mutagenic event, a phenomenon which can be mimicked by transformation with a mutated *ras* oncogene introduced by transfection (Sinha *et al.*, 1986). In this aspect they are much more amenable to the carcinogenic activity of aflatoxin than primary rat hepatocyte cultures. We were unable to transform primary cultures of hepatocytes. DNA synthesis was induced in those non-dividing primary hepatocyte cultures by Epidermal Growth Factor and they were treated with $AFB_1$ in late Gl/S-phase in a protocol similar to the transformation of BL8 cells. Primary hepatocytes, after 16 h in culture, retain sufficient endogenous metabolism of the procarcinogen to its active form, and no extrinsic metabolising system was provided. In the case of $AFB_1$ metabolism, where cytotoxicity shows a direct relationship with 8–9 epoxide formation and DNA binding, evidence of cytotoxicity in the primary hepatocyte culture was taken as an indicator of production of the ultimate carcinogen. In spite of obvious dose-dependent cytotoxicity, we could obtain no transformed cell lines as could be obtained from BL8 cells.

Previous work from this laboratory has shown that the rate of microsomal metabolism of $AFB_1$ by male quail is higher than the corresponding fraction from the rat liver and also a much higher proportion of the metabolism proceeds through 8,9-$AFB_1$ epoxidation in the quail (Neal *et al.*, 1986). It was therefore decided to use quail microsomes in preference to the S-9 metabolic activation system which has been used to activate $AFB_1$ for Ames testing and also for the transformation of C3H/10T½ cells (Billings *et al.*, 1985). Cells were treated in the late G1 and S-phase of the cell cycle which is very sensitive to the action of carcinogens (Marquardt, 1974; Milo & DiPaolo, 1978).

On treatment with carcinogens there was a dose dependent cytotoxic effect, which was much more at 5 and 10 $\mu g ml^{-1}$ $AFB_1$. This reflects the efficacy of the microsomal metabolising system because BL8 cells, not having the relevant biochemical pathways, are not susceptible to the action of the procarcinogen alone. Neoplastic transformation as seen by the property of mouse tumorigenicity was evident at higher doses (5 and 10 $\mu g ml^{-1}$), where cell survival was less than 1% of the original population.

Carcinogens can be cytotoxic by a variety of mechanisms because of the multiple ways they can cause cell death e.g. the ubiquitous macromolecular binding of $AFB_1$. Cell transformation is a more specific and rare event, reflecting the binding of the carcinogen to a site or limited number of sites in the DNA. For the carcinogen N-methyl-N′-nitro-N-nitrosoguanidine, it has been shown that while cytotoxicity is linearly related to dose, the probability of transformation among survivors increases logarithmically (Grisham *et al.*, 1980*b*). While the exact kinetics for $AFB_1$ have not been worked out, a similar behaviour would explain the detection of transformed BL8 cells at concentrations showing the highest degrees of cytotoxicity.

The tumour type produced by $AFB_1$ transformed BL8 cells is consistent with the results obtained previously by workers using cultured rat liver derived epithelial cell lines for transformation by different carcinogens. In spite of the cell lines being epithelial by established morphological and ultrastructural criteria, most investigators report the presence of sarcomatous tumours, and tumours of a mixed histopathology, and less frequently adenocarcinomas (Borek, 1980). Though BL8 cells have the capacity to form differentiated epithelial tumours (Sinha, *et al.*, submitted for publication), transformation by $AFB_1$ *in vitro* resulted in tumours of an undifferentiated histology.

GGT was induced throughout the dose range of $AFB_1$ treatment, even in doses which did not cause cell transformation. Its induction was not dose dependent and values in all sets of treated cells were uniformly higher than the parent line. We have earlier shown that GGT is induced in cells transformed by a *ras* oncogene (Sinha *et al.*, 1986). However, the strong association of GGT with tumorigenesis does not represent a critical requirement and the factors involved in the induction of the enzyme point to a cellular response rather than a change crucial to neoplasia. GGT can influence the metabolism of $AFB_1$ and may have a role in the selection of cells resistant to the toxic action of the carcinogen (Moss *et al.*, 1984; Campbell & Hayes, 1976). In this study cells surviving the carcinogen treatment had increased GGT levels even though exposure was relatively short and culture conditions did not put any selective pressure on the survivors.

### References

BERRY, M.N. & FRIEND, D.S. (1969). High-yield preparation of isolated rat liver parenchymal cells: a biochemical and structural study. *J. Cell. Biol.*, **43**, 506.

BILLINGS, P.D., HEIDELBERGER, C. & LANDOLPH, J.R. (1985). S-9 metabolic activation enhances aflatoxin mediated transformation of C3H/10T½ cells. *Toxicol. Appl. Pharmacol.*, **77**, 58.

BOREK, C. (1980). Differentiation, metabolic activation and malignant transformation in cultured liver cells exposed to chemical carcinogens. In *Advances in Modern Environmental Toxicology*, **I**. Mammalian cell transformation by chemical carcinogens, Mishra, V. *et al.* (eds) p. 297. Senate Press Inc: Princeton, N.J.

CAMPBELL, G.T. & HAYES, J.R. (1976). The role of aflatoxin metabolism in its toxic lesion. *Toxicol. Appl. Pharmacol.* **35**, 199.

FOSTER, P.L., EISENSTADT, E. & MILLER, J.H. (1983). Base substitution mutations induced by metabolically activated aflatoxin $B_1$. *Proc. Natl. Acad. Sci. USA*, **80**, 2695.

GRISHAM, J.W. (1980). Cell types in long term propagable cultures of rat liver. In: Borek, C. and Williams, G.M. (eds). Differentiation and Carcinogenesis in Liver Cell Cultures. *Ann. N.Y. Acad. Sci.*, **349**, 128.

GRISHAM, J.W., GREENBERG, D.S., KAUGMAN, D.G. & SMITH, G.J. (1980). Cell cycle related toxicity and transformation of 10T½ cells treated with N-methyl-N′-nitro-N-nitrosoguanidine. *Proc. Natl. Acad. Sci. USA*, **77**, 4813.

GUZELIAN, P.S., BISSEL, D.M. & MEYER, V.A. (1977). Drug metabolism in adult rat hepatocytes in primary monolayer cultures. *Gastroenterology*, **72**, 1232.

KALENGAYI, M.M.R., RONCHI, G. & DESMET, V.J. (1975). Histochemistry of gamma-glutamyl transpeptidase in rat liver during aflatoxin $B_1$-induced carcinogenesis. *J. Natl. Cancer Inst.*, **55**, 579.

MANSON, M.M., LEGG, R.F., WATSON, J.V., GREEN, J.A. & NEAL, G.E. (1981). An examination of the relative resistances to aflatoxin $B_1$ and susceptibilities of $\gamma$-glutamyl p-phenylene diamine mustard of $\gamma$-glutamyl transferase B negative and positive cell lines. *Carcinogenesis*, **2**, 661.

MARQUARDT, H. (1974). Cell cycle dependence of chemically induced malignant transformation *in vitro*. *Cancer Res.*, **34**, 1612.

MILO, G.E. & DIPAOLO, J.A. (1978). Neoplastic transformation of human diploid cells *in vitro* after chemical carcinogen treatment. *Nature*, **275**, 130.

MONTESANO, R., SAINT VINCENT, L. & TOMATIS, L (1973). Malignant transformation *in vitro* of rat liver cells by dimethyl nitrosamine and N-methyl-N′-nitro-N-nitrosoguanidine. *Br. J. Cancer*, **28**, 215.

MOSS, E.J., MANSON, M.M. & NEAL, G.E. (1984). Effect of manipulation of $\gamma$-glutamyl transpeptidase levels on biliary excretion of aflatoxin $B_1$ conjugates. *Carcinogenesis*, **5**, 869.

NEAL, G.E., JUDAH, D.J. & GREEN, J.A. (1986). The *in vitro* metabolism of Aflatoxin $B_1$ catalysed by hepatic microsomes isolated from control or 3-methylcholanthrene-stimulated rats and quail. *Toxicol. Appl. Pharmacol.*, **82**, 454.

NEWBERNE, P.M. & ROGERS, A.E. (1973). Animal model of human disease. Primary hepatocellular carcinoma: Aflatoxin carcinogenesis in the rat. *Am. J. Pathol.*, **72**, 137.

SELL, E. & LEFFERT, H.L. (1982). An evaluation of cellular lineages in the pathogenesis of experimental hepatocellular carcinoma. *Hepatology*, **2**, 77.

SINHA, S., MARSHALL, C.J. & NEAL, G.E. (1986). $\gamma$-glutamyltranspeptidase and the *ras*-induced transformation of a rat liver cell line. *Cancer Res.*, **46**, 1440.

SMITH, G.D., DING, J.L. & PETERS, T.J. (1979). A sensitive fluorimetric assay for gamma-glutamyl transferase. *Anal. Biochem.*, **100**, 136.

SKILLETER, D.N., PRICE, R.J. & LEGG, R.F. (1983). Specific $G_1$ S-phase cell cycle block by beryllium as demonstrated by cytofluorimetric analysis. *Biochem. J.*, **216**, 773.

WILLIAMS, G.M., ELLIOTT, J.M. & WEISBURGER, J.H. (1973). Carcinoma after malignant conversion *in vitro* of epithelial like cells from rat liver following exposure to chemical carcinogens. *Cancer Res.*, **33**, 606.